# Application of Asteraceae biomass and biofertilizers to improve potato crop health by controlling black scurf disease

**DOI:** 10.3389/fpls.2024.1437702

**Published:** 2024-09-10

**Authors:** Muhammad Rafiq, Amna Shoaib, Arshad Javaid, Shagufa Parveen, Muhammad Ahmad Hassan, Hafiz Husnain Nawaz, Chunsong Cheng

**Affiliations:** ^1^ Jiangxi Key Laboratory for Sustainable Utilization of Chinese Materia Medica Resources, Lushan Botanical Garden, Chinese Academy of Science, Jiujiang, Jiangxi, China; ^2^ Faculty of Agricultural Sciences, Department of Plant Pathology, University of the Punjab, Lahore, Pakistan; ^3^ School of Resources and Environment, Anhui Agricultural University, Hefei, China; ^4^ Faculty of Agricultural, Food and Environmental Sciences, Free University of Bozen Bolzao, Bolzano, Italy

**Keywords:** sustainable agriculture, biofertilizers, weeds, disease management, medicinal plants, antimicrobial potential

## Abstract

Potato (*Solanum tuberosum* L.) cultivation in Pakistan faces challenges, with black scurf disease caused *by Rhizoctonia solani* Kühn being a significant concern. Conventional methods like chemical fungicides partially control it, but an effective solution is lacking. This study explores the potential of biofertilizers and soil amendments from Asteraceae weed biomass to manage the disease. Two potato varieties, Karoda and Sante, were chosen, and two biofertilizers, Fertibio and Feng Shou, were tested alone or with *Xanthium strumarium* biomass. Disease pressure was highest in the positive control, with significant reduction by chemical fungicide. *X. strumarium* biomass also decreased disease incidence significantly. Fertibio showed better efficacy than Feng Shou. Physiological and biochemical attributes of plants improved with biofertilizer and biomass application. Tuber weight, photosynthetic pigments, total protein content, and antioxidant enzymes (CAT, POX, and PPO) were positively correlated. Combined application of Fertibio and S. marianum biomass effectively managed black scurf disease. These eco-friendly alternatives could enhance disease management and yield. Future research should explore their cost-effectiveness, commercialization, and safety.

## Introduction

Potato (*Solanum tuberosum* L.) ranks third globally in consumption as an important non-grain crop and first among root and tuber crops (Vollmer et al., 2017). Its significant contribution to food security and nutrition, especially in developing countries, makes it comparable to rice, wheat, and maize (Shetty et al., 2023). Potatoes are highly nutritious, easy to digest, and produced in bulk, making them a globally popular vegetable (Fernández-Ortuño et al., 2008). In 2021, global potato production reached 376 million tons, with China and India being the top producers at 94 million and 54 million tons, respectively (Munnaf et al., 2021). In Pakistan, potatoes are grown extensively, producing roughly 4.1 million tons, primarily in the central and northern plains of Punjab and KPK. Additionally, districts in Baluchistan, such as Pishin, Killa Saifullah, Kalat, and the Gilgit district in Gilgit-Baltistan, support potato production. However, Pakistan's potato production has not yet reached its maximum potential compared to neighbouring India and Bangladesh (Majeed and Muhammad, 2018).

Black scurf caused by fungal pathogen *Rhizoctonia solani* is among the one of the major constraint reducing the quality of the produced resulting in low market value eventually posing an economic threat to the income of farmers. The disease reduces tuber size and number, causes tuber deformities, and leads to sclerotial formation on tubers. Yield losses can reach up to 25% in India, 30% in Canada, and 50% elsewhere (Chaudhary et al., 2024).

Control is challenging due to the fungus's prolonged survival as sclerotia and its wide host range. As a results there is a dire need to develop a sustainable, eco-friendly approach to manage the disease. The utilization of medicinal, allelopathic, and aromatic plants against plant diseases is promising, yet less than 1% of higher plant species have been studied for their phytoactivities. Plants like those in the Asteraceae family possess antifungal properties due to various bioactive compounds, but their efficacy depends on environmental factors and the specific pathogen system (Sana et al., 2017). Phytochemicals like flavonoids and phenolics, derived from plant metabolic pathways, are abundant in leaves and fruit skin, contributing to host plant disease resistance by altering microbial cell permeability and membrane function, ultimately inhibiting enzyme activity and disrupting pathogen metabolism (El-Mogy and Alsanius, 2012). Existing literature indicated considerable antifungal activity of extracts of many Asteraceous weeds (Bandeira Reidel et al., 2018). *Xanthium strumarium*, a globally distributed Asteraceous weed, exhibits diverse biological properties, including antifungal activity, yet its potential as an agricultural fungicide remains underexplored (Haider et al., 2020). When used as soil amendments, Asteraceae biomass can improve soil structure, enhance microbial diversity, and suppress soil-borne pathogens through allelopathic interactions (Li et al., 2020). However, the biomass of Asteraceae family weeds, combined with biofertilizers, presents a promising option for plant disease management.

Biofertilizers are composed of beneficial microorganism including fungi and bacteria are cost-effective, eco-friendly, self-sustaining, and possess significant antifungal potential compared to chemical fungicides. Windisch et al. (2017) demonstrated the efficacy of *Pseudomonas jessenii* and *Serratia plymuthica* in managing bottom rot disease in lettuce caused by *R. solani*. Isolates of *Azotobacter* sp. inhibited *R. solani* growth by 53-72%, while Nontji et al. (2019) suggested the use of indigenous rhizobial microflora consortium as biological fertilizers and biopesticides against *R. solani* due to their nitrogen-fixing, phosphate-dissolving, and indole acetic acid producing capabilities. Microorganisms in commercially available biofertilizers enhance crop growth by inducing host plant resistance against specific pathogens (Saleem et al., 2022). Understanding the role of different microbial inoculants against *R. solani* is crucial for effective disease management and their application with organic soil amendments is a new approach to enhance the plant and soil health.

The combine application of plant biomass of *X. strumarium* and biofertilizers is a new way of managing the plant diseases. This study was performed to assess the effect of plant biomass of *X. strumarium* and biofertilizer in managing black scurf disease in potato through analysing morpho-growth and biochemical traits of the plants.

## Materials and methods

### Pot trial for disease management

*In planta*, a pot experiment was conducted to evaluate the disease-managing potential of dry biomass of *X. strumarium* and biofertilizers The experiment included fourteen treatments, each in triplicate, and was carried out in an open backfield located at 31.5204° N latitude, 74.3587° E longitude in Lahore, Pakistan, for 90 days in a completely randomized design (CRD). There were following treatments for *X. strumarium* biomass amendment with each potato variety.

**Treatments**

**T_1_**     − control

**T_2_**    + control (RS)

**T_3_**     Fungicide (FC)

**T_4_**    1% XSB + RS

**T_5_**    2% XSB + RS

**T_6_**    3% XSB + RS

**T_7_**    BF1 (Ferti Bio) + RS

**T_8_**    1% XSB + RS + BF1

**T_9_**    2% XSB + RS + BF1

**T_10_**    3% XSB + RS + BF1

**T_11_**    BF2 (Feng Shou) +RS

**T_12_**    1% XSB + RS + BF2

**T_13_**    2% XSB + RS + BF2

**T_14_**    3% XSB + RS + BF2

XSB: *Xanthium strumarium* plant biomass; RS: *Rhizoctonia solani*.

### Soil fumigation and pathogen inoculation

Prior seed sowing, the sandy-loam garden soil was fumigated using a 2.5% formalin solution (Yaqoob et al., 2024) and filled (7 kg pot^-1^ measuring 30 cm × 25 cm length and width. For the multiplication of fungus inoculum, boiled and autoclaved millet seeds were inoculated with the fungus discs and incubated at 30°C for 15 days. After that, fumigated soil was inoculated with colonized millet seeds (100 g/pot) containing a sclerotial count (2 × 10^7^ sclerotia/mL), and the soil was left for seven days.

### Mixing of weed biomass

*X. strumarium* is commonly found in cultivated fields, Punjab, Pakistan. Mature plants of *X. strumarium* were collected from University of the Punjab Lahore, Pakistan during June 2017 according to prescribed rules in The Pakistan Trade Control of Wild Fauna and Flora Act, 2012. The whole plants were dried, cut into small pieces and thoroughly crushed. After 7 days of pathogen inoculation in soil, dried biomass of weed was thoroughly mixed in soil of respective pots @ 0.5%, 1% and 1.5%. Pots were irrigated with tap water and left for another 7 days to homogenize the soil conditions.

### Application of fungicide

Chemical fungicide Dithane M-45 (Dithiocarbamate) was procured from the local market. It was applied at tubers before sowing at rat of 3 g Kg^-1^ seed tubers (Rauf et al., 2007).

### Procurement and application of biofertilizers

Two commercial biofertilizers *viz.* Fertibio and Feng Shou were used. Fertibio is a product of NIBGE, Pakistan and Feng Shou, a liquid bacterial culture, is manufactured by CV. Green life Bioscience, Bogor Indonesia. One hundred grams of Fertibio and 100 mL of Feng Shou were added to 1 L of sterilized water separately, and potato tubers were immersed for half an hour in these before sowing.

### Sowing of tubers

Two potato varieties *viz.* Karoda and Sante belong to susceptible and moderately susceptible groups, respectively were selected from the screening trials. Pots prepared by inoculating with pathogen and soil amendment with biofertilizers and weed biomass were used for sowing of tubers (2 tubers pots^-1^).

### Physiological tests

The physiological tests: chlorophyll, carotenoid contents, total protein contents, and antioxidants enzymes like CAT, POX and PPO of the potato leaves were estimated after 30 days of sowing as descried previously (Rafiq et al., 2024).

### Disease and yield assessment

Potato tubers were harvested after 90 days and respective data regarding disease incidence, severity and number and weight of tubers plant^-1^ were recorded.

### Statistical analysis

MS Excel program was used to calculate the standard errors of means of five replicates. Data from both laboratory and field trial was subjected to analysis of variance, after that, treatments means were separated by LSD Test with the help of computer software Statistic 8.1.

## Results

### Disease incidence and severity

**Figure 1:**
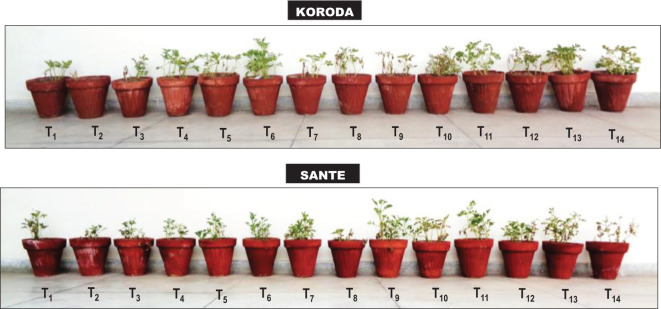
Effect of *Rhizoctonia solani* (RS), and soil amendment by *Xanthium strumarium* plant biomass (XSB) and two commercial biofertilizers on plant growth and black scurf disease of potato. T_1_: − control; T_2_: + control (RS); T_3_: 0.5% XSB + RS; T_4_: 1% XSB + RS; T_5_: 1.5% XSB + RS; T_6_: Fungicide (FC) + RS; T_7_: BF1 (Fertibio) + RS; T_8:_ 0.5% XSB + RS + BF1; T_9_: 1% XSB + RS + BF1; T_10_: 1.5% XSB + RS + BF1; T_11_: BF2 (Feng Shou) + RS; T_12_: 0.5% XSB + RS + BF2; T_13_: 1% XSB + RS + BF2; T_14_: 1.5% XSB + RS + BF2.

**Figure 2:**
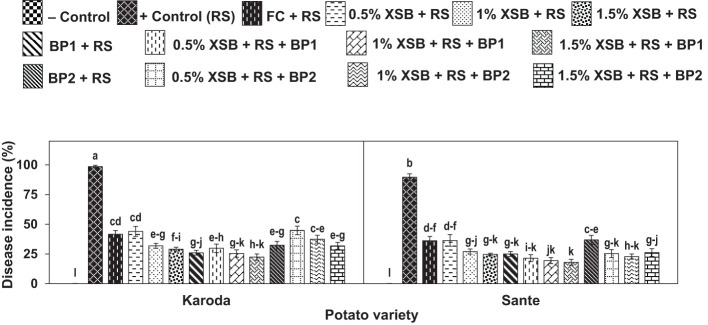
Effect of soil amendment with *Xanthium strumarium* plant biomass (XSB), and two commercial biofertilizers namely Fertibio (BF1) and Feng Shou (BF2) on incidence of black scurf disease of potato in *Rhizoctonia solani* (RS) inoculated soil. Vertical bars show standard errors of means of five replicates. Values with different letters at their top show significant difference (*P* ≤ 0.05) as determined by LSD Test.

No disease was observed in negative control (without *R. solani*) in either of the two potato genotypes. The highest disease occurrence was observed in positive control (with *R. solani* only) of Karoda and Sante with disease incidence 100% and 90%, and disease severity rating (DR) 5 and 4, respectively. Chemical fungicide significantly reduced disease incidence to 44% (DR: 3.75) and 36% (DR: 3.50) in Karoda and Sante, respectively. Application of *X. strumarium* plant biomass (XSB) as well as Biofertilizers (BF1 and BF2) significantly and variably managed disease as compared to positive control of both varieties. However, application of 3% plant biomass alone and in combination with BF1 showed the highest management of black scruf disease. Different doses of XSB significantly decreased disease incidence to 29–44% (DR 3.00–3.75) in Karoda, and to 25–36% (DR 3.50–2.75) in Sante. In Karoda, application of BF1 and XSB + BF1 significantly decreased disease incidence to 26% and 22%, respectively. Efficacy of BF2 in controlling black scruf disease incidence (32%) did not differ significantly either of their combined effect with 2% and 3% plant biomass. In Sante, BF1 or 1–3% XSB + BF1 significantly decreased disease incidence to 22% ± 3. However, BF2 application resulted in 37% disease incidence, the disease pressure decreased significantly decreased to 25% due to combined effect of BF2 and different doses of (1–3%) plant biomass (XSB) (**Figures 1**, **2**, **Table 1**).

**Table 1:** Disease rating scale for assessment of black scurf disease on two potato verities (90 DAS).

Potato variety			
Karoda		Sante	
Treatments	Disease rating	Treatments	Disease rating
**T_1_**− control	**0.00**	**T_1_**− control	**0.00**
**T_2_** + control (RS)	**5.00**	**T_2_** + control (RS)	**4.00**
**T_3_** Fungicide (FC)	**3.75**	**T_3_** Fungicide (FC)	**3.50**
**T_4_** 0.5% XSB + RS	**3.75**	**T_4_** 0.5% XSB + RS	**3.50**
**T_5_** 1% XSB + RS	**3.50**	**T_5_** 1% XSB + RS	**3.00**
**T_6_** 1.5% XSB + RS	**3.00**	**T_6_** 1.5% XSB + RS	**2.75**
**T_7_** BF1 (Fertibio) + RS	**3.25**	**T_7_** BF1 (Fertibio) + RS	**3.00**
**T_8_** 0.5% XSB + RS + BF1	**3.50**	**T_8_** 0.5% XSB + RS + BF1	**3.25**
**T_9_** 1% XSB + RS + BF1	**3.25**	**T_9_** 1% XSB + RS + BF1	**3.00**
**T_10_** 1.5% XSB + RS + BF1	**3.25**	**T_10_** 1.5% XSB + RS + BF1	**3.00**
**T_11_** BF2 (Feng Shou) +RS	**3.75**	**T_11_** BF2 (Feng Shou) +RS	**3.50**
**T_12_** 0.5% XSB + RS + BF2	**3.75**	**T_12_** 0.5% XSB + RS + BF2	**3.50**
**T_13_** 1% XSB + RS + BF2	**3.50**	**T_13_** 1% XSB + RS + BF2	**3.50**
**T_14_** 1.5% XSB + RS + BF2	**3.50**	**T_14_** 1.5% XSB + RS + BF2	**3.25**

XSB, Xanthium strumarium plant biomass; RS, Rhizoctonia solani.

0-5 rating scale where 0= No symptom; 1= less than 1%; 2 = 1-10%; 3 = 11-20%; 4 = 21-50%; and 5 = 51% or more tuber area affected.

Bold values show the severity of the disease in specific treatment and variety.

### Tuber growth attributes

**Figure 3:**
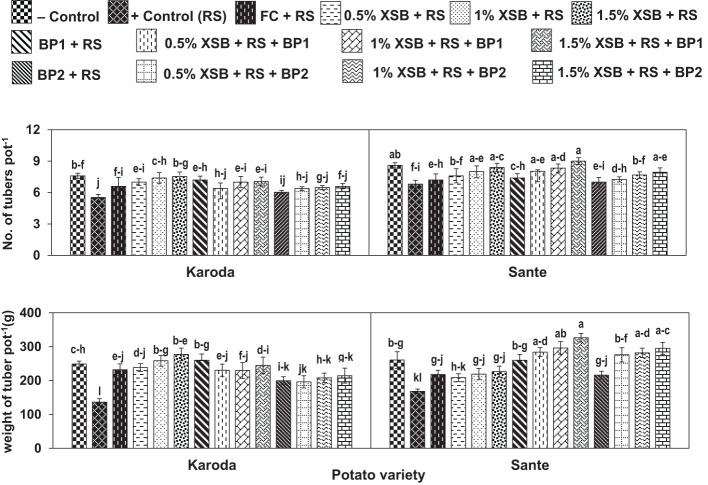
Effect of soil amendment with *Xanthium strumarium* plant biomass (XSB), and two commercial biofertilizers namely Fertibio (BF1) and Feng Shou (BF2) on tuber growth of potato in *Rhizoctonia solani* (RS) inoculated soil. Vertical bars show standard errors of means of five replicates. Values with different letters at their top show significant difference (*P* ≤ 0.05) as determined by LSD Test.

Number of tubers pot^-1^ were significantly decreased by 27% and 21% in Karoda and Sante, respectively due to effect of *R. solani* inoculation in positive control as compared respective negative control treatment (**Figure 3**). In Karoda, number of tubers pot^-1^ were improved significantly by 20% due to application of fungicide over positive control. However, use of *X. strumarium* plant biomass in different doses (1–3%) resulted in more improvement in tuber number than chemical fungicides causing significant increase of 27-37% over positive control. Applying BFI or XSB + BF1 in *R. solani* inoculated soil also significantly increased tubers number pot^-1^ in Karoda by 23–30% as compared to positive control. In case of Sante, only 2% and 3% XSB, and their combination with BF1 significantly improved number of tubers pot^-1^ by 18–32% as compared to positive control. BF2 or XSB + BF2 failed to improve the said attribute significantly under *R. solani* stress over positive control (**Figure 3A**).

Infection caused by *R. solani* resulted in significant reduction in weight of tubers by 45% and 37% in comparison to negative control in Koroda (249 g) and Sante (261 g), respectively (**Figure 3B**). Application of chemical fungicide significantly improved the said attributes by 70% and 30% in Karoda and Sante, respectively as compared to their respective positive control treatments. In Karoda, application of XSB, BFI or XSB + BF1 in *R. solani* inoculated soils, showed the highest improvement (75–104%, 91% or 69–79%) in weight of tubers. BF2 or XSB + BF2 treatments also significantly enhanced the weight of tuber under stress of *R. solani* by 44–57% with respect to positive control treatment. In Sante, all the treatments ameliorated weight of tuber to variable extents over positive control. The synergistic effect of using XSB + BF1 or XSB + BF2 was better than separate applications of either XSB or biofertilizer. Thus, significant increase of 69–94% and 64–76% was recorded in weight of tubers due to combined application of different doses of XSB with BF1 and BF2 over positive control, respectively (**Figure 3B**).

### Physiological attributes

#### Total chlorophyll and Carotenoid contents

**Figure 4:**
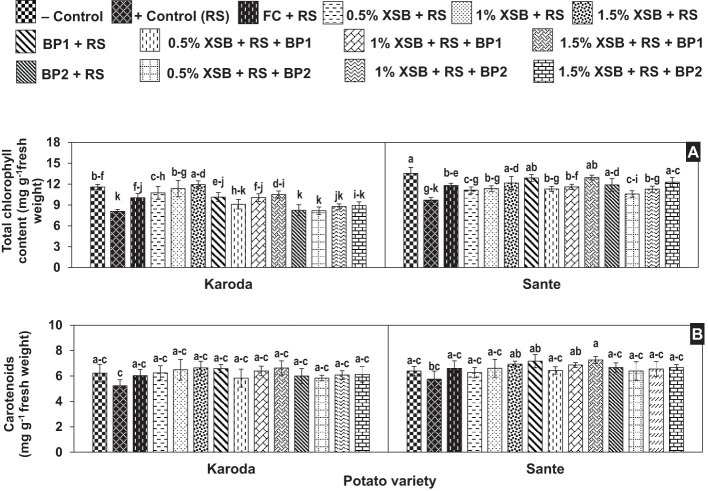
**(A, B)** Effect of soil amendment with *Xanthium strumarium* plant biomass (XSB), and two commercial biofertilizers namely Fertibio (BF1) and Feng Shou (BF2) on photosynthetic pigments of potato in *Rhizoctonia solani* (RS) inoculated soil. Vertical bars show standard errors of means of five replicates. Values with different letters at their top show significant difference (*P* ≤ 0.05) as determined by LSD Test.

The total chlorophyll content of *R. solani* inoculated plants (positive control) was significantly lower (8.10 and 9.70 mg g^-1^) than obtained (11.60 and 13.60 mg g^-1^) from non-inoculated (negative control) plants in Karoda and Sante, respectively. The total chlorophyll content of fungicides treated plants were significantly higher as compared to positive control in either variety, therefore the said trait improved by 23% in both varieties. Soil amendment with all the three doses (1, 2 and 3%) of XSB proved effective in significantly enhancing total chlorophyll content by 33–48%. However, BP1 alone or in combination with 3% XSB also showed significant improvement (28%) in the investigated attribute. No significant increase was observed in total chlorophyll content of Karoda due to the effect of other soil amendment treatments with respect to positive control. In Sante, application of either 3% XSB, BF1 or BF2 either alone or in combination with 3% XSB showed the same effect on total chlorophyll content resulting in significant improvement of 28% over positive control. The effect of other treatments on this parameter was insignificant as compared with positive control (**Figure 4A**).

The carotenoid content (6.20 and 5.80 mg g^-1^) were insignificantly decreased in positive control treatments of Karoda and Sante, respectively over negative control, while plant biomass and biofertilizers also caused insignificant improvement in the said attribute in both the varieties as compared to respective positive control (**Figure 4B**).

#### Total protein content

**Figure 5:**
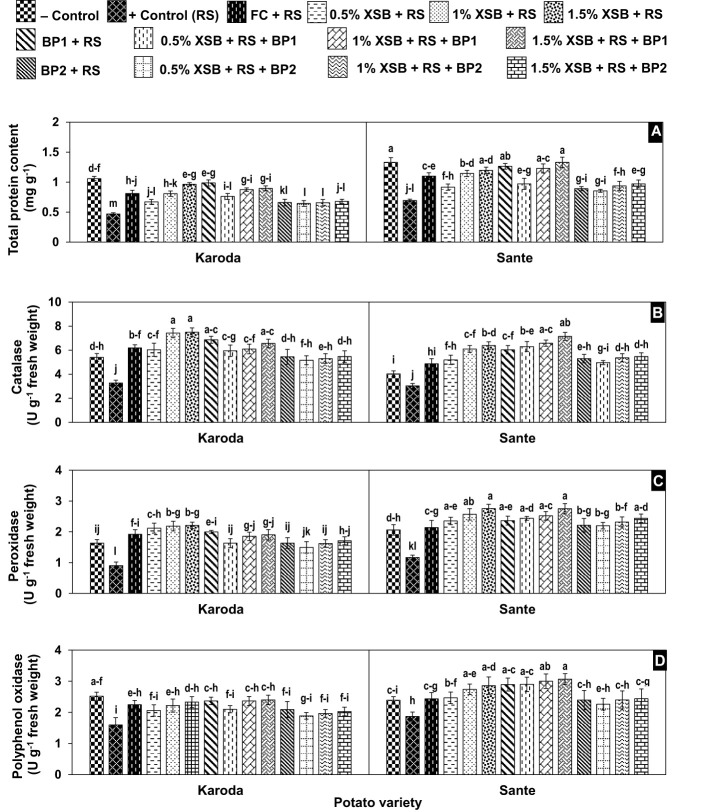
**(A-D)** Effect of soil amendment with *Xanthium strumarium* plant biomass (XSB), and two commercial biofertilizers namely Fertibio (BF1) and Feng Shou (BF2) on biochemical attributes of potato in *Rhizoctonia solani* (RS) inoculated soil. Vertical bars show standard errors of means of five replicates. Values with different letters at their top show significant difference (*P* ≤ 0.05) as determined by LSD Test.

The pathogen infection resulted in significant reduction of 56% and 48% in total protein content in Karoda and Sante, respectively when compared with their respective negative controls. Application of chemical fungicide caused significant increase of 72% and 59% in the total protein content in Karoda and Sante over their corresponding positive control, respectively. Application of *X*. *strumarium* plant biomass as soil amendment gradually increased this attribute by 43–104% (Karoda) and 32–73% (Sante) as compared to respective positive control with an increase in dose of the plant biomass. In general, within Karoda and Sante, the improvement of 86–110% and 77–92% in aforesaid attribute, respectively was statistically identical in treatments BF1, 2% XSB + BF1 and 3% XSB + BF1, while this improvement was also statistically equal to 2% XSB and 2% XSB. Likewise, BF2 as well as in combination with 1–3% doses of plant biomass exhibited comparable enhancement of 40–45% and 23–40% in the said trait in Karoda and Sante, respectively as compared to respective positive control, however, this improvement was significantly less than recorded with rest of biofungicides (**Figure 5A**).

### Catalase activity

Soil inoculation with *R. solani* resulted in a significant reduction in CAT activity (3.30 and 3.00 U g^-1^ fresh weight) of infected plant as compared to non-inoculated healthy control (5.40 and 4.00 U g^-1^ fresh weight) of Karoda and Sante, respectively. Application of chemical fungicide, plant biomass and biofertilizers significantly increased the CAT activity to variable extents in both varieties. Similar to the results reported for the total protein content, dry weed biomass without BFI as well as in combination BFI significantly enhanced CAT activity by 82–130% in Karoda and 99–136% in Sante over respective positive control. BF2 either alone or in combination with dry weed biomass significantly improved this attributes in Karoda (62%) and Sante (70%) over respective positive control (**Figure 5B**).

### Peroxidase activity

The highest POX activity (0.90 and 1.20 U g^-1^ fresh weight) was recorded in pathogen treated plants in both Karoda (1.60 U g^-1^ fresh weight) and Sante (2.10 U g^-1^ fresh weight). The effect of dry weed biomass, biofertilizers and their combination was generally similar to the effect of these amendments on CAT activity. Thus significant improvement of 70–145% and 80–136% was recorded due to effect of different amendments in Karoda and Sante over corresponding positive control, respectively (**Figure 5C**).

### Polyphenol oxidase activity

The highest reduction (37% and 22%) in PPO activity was observed in pathogen-infected samples with respect to relevant negative control in Karoda and Sante, respectively. The highest PPO activity was recorded in the range of 2.40–2.40 U g^-1^ fresh weight (Karoda) and 2.90–3.10 U g^-1^ fresh weight (Sante) in treatments including negative control, FC, 2% XSB, 3% XSB, BF1 and XSB + BF1. In rest of the treatments, PPO activity was increased insignificantly in either variety as compared to relevant positive control (**Figure 5D**).

## Discussion

Black scurf disease, caused by *R. solani*, is a widespread threat to potato worldwide, causing significant yield losses. Traditional methods struggle to manage it due to the pathogen's soil-borne nature and persistent sclerotia formation. Combining Asteraceous weeds with biofertilizers shows promise in effectively combating the disease, offering a cost-effective, biodegradable, and non-toxic solution for sustainable potato cultivation. The dried plant biomass of *X. strumarium* was used alone as well as in combination with two commercially available biofertilizers namely Fertibio and Feng Shou to evaluate their disease managing potential in Karoda (moderately susceptible) and Sante (susceptible) potato varieties. Results on the disease, growth (health markers) and physiology (stress markers) were compared with negative control, positive control and with the treatments provided with a chemical fungicide (dithiocarbamate). The highest disease pressure was observed in positive control (with *R. solani* only) of Karoda and Sante with disease incidence 100% and 90% equated to the highest sclerotia infection score (disease rating: DR: 5 and 4, respectively). Chemical fungicide significantly reduced black scurf disease incidence i.e. 44% (DR: 3.75) and 36% (DR: 3.5) in Karoda and Sante, respectively. Application of the two biofertilizers, and dry plant biomass of *X. strumarium* (XSB) significantly managed disease as compared to positive control in both the varieties. Different doses (1, 2 and 3%) of *X.* strumarium biomass significantly decreased disease incidence to 29–44% (DR 3.00–3.75) in Karoda, and to 25–36% (DR 3.50–2.75) in Sante. Likewise, in other many studies plant dry biomass was used as soil amendment to successfully manage different diseases. Sana et al. (2016) reported that increase in dose of dry leaf biomass (2% and 3%) of *Eucalyptus camaldulensis* significantly decreased incidence of collar rot in. Charcoal rot disease caused by sclerotial forming *Macrophomina phaseolina* has been managed in *Vigna mungo* and *Vigna unguiculata* by soil mixing with 1–3% dry leaf biomass of *Sisymbrium irio* and *Azadirachta indica*, respectively (Javaid et al., 2017a; Shoaib et al., 2018a; Shoaib et al., 2018b). Banaras et al. (2020) reported that soil amendment with dry biomass of an Astercaeous weed *Sonchus oleraceous* completely controlled charcoal rot of urdbean caused by *M. phaseolina.* Besides, liberation of toxic nitrogenous compounds or organic acids upon decomposition of plant biomass in potted soil could be ascribed to disease management potential of plant biomass. Occurrence of sulphur containing compounds in plant families including Asteraceae has been documented as an alternative of fumigant methyl bromide. Hence, antimicrobial and antioxidant activities of sulphur containing compounds e.g. sulphate flavonoids might be associated with activation of plant defense system against the pathogen (Teles et al., 2018), enhancement in host resistance and conservation of root system function. Broad range of antimicrobial activity of asteraceous terpenoids and flavonoids has also been confirmed (Sulsen et al., 2017).

Biofertilizer Fertibio more effectively alleviated stress of black scurf as compared to Feng Shou. Application of Fertibio resulted in disease incidence of 26% (DR: 3.25) and 25% (DR: 3.00), while Feng Shou found effective in reducing disease incidence to 32% and 37% as compared to positive control in Karoda and Santa, respectively. The disease management potential of *X. strumarium* biomass in general did not improve significantly in combination either with Fertibio or Feng Shou in both the varieties. Fertibio contained consortium of *Azospirillum*, *Azotobacter*, *Pseudomonas*, while Feng Shou had mixture of *Azospirillum* and *Azotobacter* along with other plant growth hormone producing bacteria. Previous studies have proved that biofertilizers containing different microbes are effective in managing the plant diseases either alone (Itelima et al., 2018) or in combination with different organic amendments (Javaid et al., 2017b, a). The disease managing potential of these biofertilizers might be due to improvement in plant growth through production of plant growth regulators and antibiotics, decomposition of organic matter, solubilization of phosphates and nitrogen fixation (Itelima et al., 2018). In addition, these microbes also show synergistic and antagonistic behaviour towards other microbes in the soil. The low disease managing potential of biofertilizer Feng Shou might be due to competition of bacteria in biofertilizer with soil indigenous microflora.

Growth (tuber’s number and weight), physiological (total chlorophyll conent and total protein content) and biochemical (CAT, POX and PPO) attributes were significantly decreased by 30–60% and 20–50% in positive control of Karoda and Sante, respectively, as compared to non-inoculated healthy plants in negative control. Tubers were heavily infected by dark brown to black sclerotial mass in positive control of both potato varieties. Many authors have reported down-graded tuber quality by tuber-borne sclerotia, which also caused malformed tubers with reduction in number, weight and marketable tuber yield (Jager et al., 1991; Daami-Remadi et al., 2008). Negative effect of *R. solani* has been linked with production of some phenolic and glucosidic phytotoxic substances in the infected plant parts (Daami-Remadi et al., 2008), which might cause abnormal changes in plant health and stress markers (Awan et al., 2018; Yaqoob et al., 2019). The exaggerated growth responses in tubers were visible only when the pathogen infection has disrupted intercellular communication. Alternation in bioenergetics leads to abnormal metabolism. Since *R. solani* infection caused a reduction in leaf chlorophyll content which might ascribed to inhibition of photophosphorylation by fungal toxins (Lubaina and Murugan, 2013), loss in leaf photosynthetic area promoting less light absorption, and chloroplast disorders during pathogen infection (Radwan et al., 2008). Moreover, accumulation of reactive oxygen species (ROS) after pathogen infection causes inactivation/oxidation of pre-existing pigments in chloroplasts (Lobato et al., 2010). Carotenoids remained unaffected due to effect of *R. solani* in either variety. Some authors also reported that lowering of chlorophyll a to chlorophyll b ratio indicates photoinhibition due to damage of photosystem II (Negrão et al., 2017). Furthermore, the changes in the total chlorophyll to carotenoids ratio may be one of the symptoms of oxidative stress (Grzeszczuk et al., 2018). Reduction in protein content of infected plant may direct protein denaturation or its utilization by host during defense mechanism (Lubaina and Murugan, 2013). It has been demonstrated that potato plants respond to infection by virulent *R. solani* strains by systemic activation of an array of defense genes such as of chitin-hydrolyzing enzymes and 1,3,-β-glucanase which are involved in hydrolyzing fungal cell walls (Lehtonen et al., 2008) as well as POX, PPO and CAT (defense related gene) (Riaz et al., 2023) so that plant disease resistance reactions are stimulated for preventing the invasion of pathogens. In the present study, enzyme activity decreased significantly in positive control treatments might be indicative of induction of the oxidative stress exerted by ROS and failure of plant to cope up with stress (Narayan et al., 2017).

Plant growth, physiological and biochemical attributes were significantly stimulated due to application of fungicide, *X. strumarium* biomass, Fertibio and Feng Shou up to 100, 180, 130 and 70%, respectively. Fertibio in combination with *X. strumarium* biomass showed more promising results on the said attributes. The current results were in concordance with many previous findings, where plant growth, physiology and biochemical attributes were improved significantly due to soil amendment with plant dry biomass alone or in combination with different kind of beneficial microorganism (Javaid et al., 2017a; Javaid et al., 2017b; Javaid et al., 2018a; Javaid et al., 2018b; Shoaib et al., 2018a; Shoaib et al., 2018b). Yossen et al. (2011) showed that combination of green manure and biocontrol agents (*Bacillus subtilis* or *Trichoderma harzianum*) promoted potato yield and reduced scab incidence. Increase in tuber weight and reduction in incidence of potato common scab has also been reported previously due to application of antagonistic bacterial strain *Streptomyces violaceusniger* in pot and field (Sarwar et al., 2019). The bio/organic amendments serve as bioprotective agents which not only prevent pathogen’s growth and development but could also induce resistant in potato plant against *R. solani* resulting in disease reduction (Akladious et al., 2015). Kamalakannan et al. (2004) demonstrated that in addition to direct antagonism, the biocontrol agents also increase the activity of various defense–related enzymes and chemicals in response to pathogen infection. Akladious et al. (2015) found enhancement in the activities of antioxidant enzymes after application of extract of *Ocimum basilicum* prevented *F. oxysporum* wilt disease in tomato through activation of defense mechanism associated with resistance. García-Limones et al. (2009) reported that the augmented antioxidant enzymes activities including CAT were associated with resistance to Fusarium wilt in chickpea. It was apparent n the present study that in potato leaves the activities of CAT and POX were enhanced to a higher extent in all treatments than PPO activities. POX and CAT being ROS scavenging enzymes would facilitate plant to maintain balance of ROS (Khan et al., 2018). POX along with PPO help to hinder pathogen spread through the formation of polymerized phenolic barriers around the sites of infection and trigger the synthesis of antinutritive, antibiotic, and cytotoxic compounds leading to enhanced resistance against pathogens (Smit and Dubery, 1997; Li and Steffens, 2002; Siddaiah et al., 2017). Earlier improvement in the activity of PPO was reported to be due to either solubilization of polyphenolases from cellular compartments or activation of latent PPO (Jyosthna et al., 2004).

The efficient antioxidant response in potato plant will be helpful in regulation of photosynthetic carbon reduction and protection of chloroplast from oxidative damage. Besides, improvement in carotenoid pigments might be associated with its protective role for chlorophylls from photo-oxidative destruction and degradation, where they function as energy carriers and photo-oxidation protectors (Abbas and Akladious, 2013).

## Conclusion

It was concluded that organo-bio consortia could help to alleviate the black scurf disease in potato by inducing resistance in plant through increasing activities of defense related enzymes, which reduced disease severity and improved potato marketable tuber’s attributes.

## Data Availability

The raw data supporting the conclusions of this article will be made available by the authors, without undue reservation.
